# Artificial Intelligence for the Automatic Diagnosis of Gastritis: A Systematic Review

**DOI:** 10.3390/jcm13164818

**Published:** 2024-08-15

**Authors:** Daria Claudia Turtoi, Vlad Dumitru Brata, Victor Incze, Abdulrahman Ismaiel, Dinu Iuliu Dumitrascu, Valentin Militaru, Mihai Alexandru Munteanu, Alexandru Botan, Dan Alexandru Toc, Traian Adrian Duse, Stefan Lucian Popa

**Affiliations:** 1 Faculty of Medicine, “Iuliu Hatieganu” University of Medicine and Pharmacy, 400000 Cluj-Napoca, Romania; turtoidariaclaudia@gmail.com (D.C.T.); vicincze@yahoo.com (V.I.); botan.alexandru@elearn.umfcluj.ro (A.B.); adrianduse@yahoo.com (T.A.D.); 2 2nd Medical Department, “Iuliu Hatieganu” University of Medicine and Pharmacy, 400000 Cluj-Napoca, Romania; abdulrahman.ismaiel@yahoo.com (A.I.); popa.stefan@umfcluj.ro (S.L.P.); 3 Department of Anatomy, “Iuliu Hatieganu” University of Medicine and Pharmacy, 400000 Cluj-Napoca, Romania; d.dumitrascu@yahoo.com; 4 Department of Internal Medicine, Clinical Municipal Hospital, 400139 Cluj-Napoca, Romania; valentin.militaru@umfcluj.ro; 5 Department of Medical Disciplines, Faculty of Medicine and Pharmacy, University of Oradea, 410087 Oradea, Romania; mihaimunteanual@yahoo.com; 6 Department of Microbiology, “Iuliu Hatieganu” University of Medicine and Pharmacy, 400000 Cluj-Napoca, Romania; toc.dan.alexandru@elearn.umfcluj.ro

**Keywords:** gastritis, artificial intelligence, automatic diagnosis, gastroenterology

## Abstract

**Background and Objective**: Gastritis represents one of the most prevalent gastrointestinal diseases and has a multifactorial etiology, many forms of manifestation, and various symptoms. Diagnosis of gastritis is made based on clinical, endoscopic, and histological criteria, and although it is a thorough process, many cases are misdiagnosed or overlooked. This systematic review aims to provide an extensive overview of current artificial intelligence (AI) applications in gastritis diagnosis and evaluate the precision of these systems. This evaluation could highlight the role of AI as a helpful and useful tool in facilitating timely and accurate diagnoses, which in turn could improve patient outcomes. **Methods**: We have conducted an extensive and comprehensive literature search of PubMed, Scopus, and Web of Science, including studies published until July 2024. **Results**: Despite variations in study design, participant numbers and characteristics, and outcome measures, our observations suggest that implementing an AI automatic diagnostic tool into clinical practice is currently feasible, with the current systems achieving high levels of accuracy, sensitivity, and specificity. Our findings indicate that AI outperformed human experts in most studies, with multiple studies exhibiting an accuracy of over 90% for AI compared to human experts. These results highlight the significant potential of AI to enhance diagnostic accuracy and efficiency in gastroenterology. **Conclusions**: AI-based technologies can now automatically diagnose using images provided by gastroscopy, digital pathology, and radiology imaging. Deep learning models exhibited high levels of accuracy, sensitivity, and specificity while assessing the diagnosis, staging, and risk of neoplasia for different types of gastritis, results that are superior to those of human experts in most studies.

## 1. Introduction

Gastritis is a gastric inflammatory disease associated with injury to the mucosa. Given that most people with microscopic stomach inflammation are asymptomatic, it is a histological entity, not a clinical one [1]

The disease can be caused by infectious agents, alcohol abuse, medication, autoimmune diseases, bile reflux, and stress. Worldwide, the incidence of gastritis matches demographic data such as a population’s social and economic status. The epidemiology of gastric inflammation overlays that of *Helicobacter pylori* (HP) infection, which affects approximately half of the world’s population [2].

Gastritis is classified and graded using the Updated Sydney System, which divides gastric inflammation depending on topography, morphology, and etiology. Accordingly, there are three main types of gastritis: acute, chronic, and special forms [3]. Staging of gastritis is performed using the Operative Link on Gastritis Assessment (OLGA) with the help of atrophy score and topography, both of which are determined by biopsy during an upper gastrointestinal (UGI) tract endoscopy [4].

The diagnosis of gastritis is based on clinical, endoscopic, and histological criteria. Patients with gastritis may present dyspeptic syndrome, anemic syndrome, or UGI bleeding, or they may be asymptomatic. UGI endoscopy, which is mandatory for diagnosing gastritis alongside the biopsy, offering the histopathological report, is the gold standard for diagnosing the inflammation of the gastric mucosa [5].

Artificial intelligence (AI) has been tremendously researched in the medical field in the past decade to assess whether it could improve diagnosis accuracy. Deep learning (DL) methods are one of the most exploited machine learning methods, providing the best abilities to extract visual details from images, even undetected ones for humans, and to examine a great quantity of information in a short time [6]. Especially in gastroenterology and hepatology, which significantly depend on large imaging studies, AI is rapidly emerging as a transformative tool providing considerable potential for augmenting diagnostic and treatment processes. Multiple AI systems, meticulously trained on extensive databases and labeled endoscopic images, exhibit remarkable abilities in recognizing subtle mucosal lesions like polyps or early signs of malignancy [7]. Moreover, AI can be utilized for real-time image analysis during endoscopies, offering immediate insight to the gastroenterologist and guiding therapeutic procedures with increased accuracy [7].

The importance of the addressed topic lies in the fact that facile and early diagnosis by combining artificial and human intelligence could considerably improve the prognosis and evolution of this pathology, since chronic gastritis represents one of the initial stages of the gastric carcinogenesis process. Untreated, acute gastritis will evolve into chronic gastritis, which can give rise to precancerous lesions such as atrophic gastritis, dysplasia, and metaplasia, leading to gastric adenocarcinoma [8].

Consequently, this systematic review aims to offer an extensive overview of actual AI applications and analyze these systems’ precision to diagnose gastritis automatically. Our review provides a comprehensive analysis of the several AI techniques used in diagnosing gastritis, evaluating their efficacy and pointing out significant developments and drawbacks. By combining the results of several investigations, we give a coherent summary showing the various applications of AI and how they affect the precision of diagnoses.

## 2. Materials and Methods

This systematic review was carried out according to the PRISMA guidelines for systematic reviews. The protocol for this systematic review was registered in INPLASY (registration number: INPLASY202470095). PubMed, Cochrane Library, EMBASE, and WILEY databases were thoroughly screened for significant publications concerning the use of AI in the automatic diagnosis of gastritis. The relevant search of words included gastritis AND (artificial intelligence OR deep learning OR neural network OR machine learning OR computer-aided diagnosis OR automated diagnosis OR automated gastric endoscopy OR digital pathology OR automated ultrasound OR automated X-ray). Exclusion criteria included studies written in a language other than English, abstracts, animal studies, pediatric studies, case reports, editorials, letters to the editor, and conference publications.

**Figure 1:**
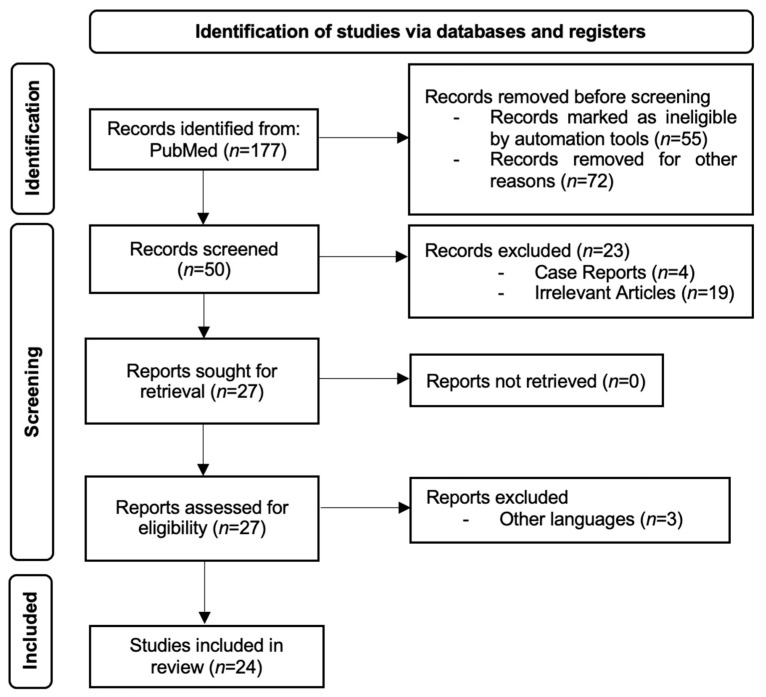
PRISMA flow diagram for study selection.

Two independent authors (S.L.P. and V.D.B.) examined eligibility, titles, abstracts, and the full text of articles that fit all criteria. Data extraction was also performed individually by both assessors. Retrieved data, covering the authors’ names, publishing year, country or study population, sample size, study design, the technique used to diagnose gastritis, and AI application, was analyzed. Figure 1 displays the search algorithm utilizing the PRISMA flow diagram.

The initial search identified 177 studies. We screened 50 studies and excluded 26 articles due to being irrelevant to the addressed topic (*n* = 19), other languages (*n* = 3), and case reports (*n* = 4). Lastly, 24 studies met our inclusion and exclusion criteria and were included in our systematic review, as shown in Figure 1.

## 3. Results

The gold standard for diagnosing any type of gastritis, especially the chronic ones, is the histopathological analysis of the tissular fragments biopsied during the UGI endoscopy. Even though there are serological markers, such as the levels of pepsinogen, gastrin-17, and the anti-parietal gastric cell antibodies, that could be used to screen the patients before the invasive procedure, there are currently no tests with adequate specificity to replace the standard diagnostic procedure [9].

Chronic atrophic gastritis (CAG) is the most frequent precancerous lesion in the gastric carcinogenesis pathway. The risk for intestinal-type adenocarcinoma increases with the severity of the atrophy [9]. In Asian countries with a high prevalence of gastric cancer, such as Japan and South Korea, endoscopic screening protocols have been implemented to identify the malignancy from the early stages so that the patients have a higher chance of recovery. However, the endoscopy remains an invasive procedure with a risk of bleeding, pain, and discomfort during the procedure [10].

AI has been extensively exploited in the medical field in the last decade to assess its potential aid in improving clinicians’ skills in arriving at a diagnosis. DL algorithms could be put into practice to diagnose different types of gastritis to reduce the number of endoscopies and biopsies made to evaluate the disease, shorten the diagnosis time, reduce human-made errors, reduce the workload of endoscopists and pathologists, improve the abilities of young clinicians, and identify benign and malign lesions rapidly [11].

Diagnosis of gastritis based on endoscopic images, pathology studies, double-contrast UGI barium X-rays, and even clinical and serological findings made by the DL methods could serve as a tool to help clinicians make diagnostic decisions. Most AI models can provide heat maps, which can accentuate certain areas by color-coding them, providing a visual aid to simplify the diagnosis process.

### 3.1. Artificial Intelligence Based on Endoscopic Images

The principal studies researching AI effectiveness in recognizing gastritis on endoscopic images and/or videos are shown in Table 1.

Shi et al. developed a new DL model to aid the diagnosis of CAG, using 10,961 endoscopic images and 118 video clips from three datasets: an internal one, an external one, and a video test set. Firstly, they compared four DL networks: two transformers and two CNNs. Then, they chose the network that outperformed the others and added to it the global attention mechanism (GAM). The final, improved CAG recognition model was GAM-EfficientNet, with an F-score of 94,26%. Furthermore, they tested it against three trained endoscopists to evaluate its performance. For this AI model, the sensitivity, specificity, and accuracy scored 93%, 94%, and 93.5% for the external test set and 96.23%, 89.23%, and 92.37% for the video set test, which were greater than those of the endoscopists. Additionally, this model can generate heatmaps based on the areas of interest it uses to make the diagnosis decisions, providing, in this way, a visual aid for endoscopists. Further testing and training of the model is needed to improve the diagnosis of the CAG and classify the severity of the atrophy [12].

Jhang et al. investigated a gastric section correlation network (GSCNet) for diagnosing the corpus-predominant gastritis index (CGI) using data from 304 patients with white-light endoscopic images of the antrum, body, and cardia (68 patients with CGI and 90 patients without CGI in the training set and 20 patients with CGI and 48 patients without CGI in the testing set). The model performed better than all three neural network methods (GoogLeNet, ResNet-50, and DenseNet-121) and two transformer methods (BoTNet and ViT) it was compared to, with an AUC of 0.984, accuracy of 0.957, sensitivity of 0.938, and specificity of 0.962 [13].

Tao et al. developed an AI system for diagnosing gastric atrophy (GA) and risk stratification. The model was trained on three data sets. The first data set comprised 453 GA images and 416 non-GA images, with 251 images having only GA and 275 also having IM. Data set 2 had 78 GA videos from 43 patients and 41 non-GA videos from 27 patients. Data set 3 was used for risk stratification and contained 20 non-GA cases and 31 mild, 30 moderate, and 21 severe endoscopic GA cases. In the image competition, the model had 0.925 accuracy, 0.927 sensitivity, 0.923 specificity, 0.929 PPV, and 0.92 NPV, obtaining a better performance than the novice endoscopists (sensitivity, accuracy, PPV, NPV), the senior endoscopists (sensitivity, accuracy, NPV), and the average endoscopists (sensitivity, accuracy, NPV), while having a worse performance than the expert endoscopists (specificity, PPV). In the video competition, the model had 0.941 accuracy, 0.949 sensitivity, 0.927 specificity, 0.961 PPV, and 0.905 NPV, better performance in sensitivity, accuracy, and NPV than all endoscopist groups. The model also had a higher accuracy than all groups of endoscopists in CAG stratification [14].

A real-time video monitoring model based on U-Net DL was developed for diagnosing CAG and OLGA during endoscopies using data from 907 patients (262 patients in the CAG group and 645 patients in the CNAG group). A total of 5290 images were used from 1711 patients (4175 images were labeled as CAG and 1115 images were labeled as CNAG; 3703 images were included in the training set with a fivefold cross for validation, and 1587 images were used to test the model). The model performed better than the endoscopists for all parameters: sensitivity (0.893 vs. 0.676), specificity (0.905 vs. 0.702), PPV (0.904 vs. 0.694), NPV (0.894 vs. 0.684), and accuracy rate (0.899 vs. 0.689). The model also performed better in diagnosing OLGA [15].

Yang et al. conducted a study to assess whether a novel DL model could detect gastric intestinal metaplasia (GIM) and CAG with an accuracy comparable to human experts. The dataset included 21,420 endoscopic images using white-light imaging (WLI) and linked-color imaging (LCI), annotated and validated by four radiologists. The AI model was equipped with the local attention grouping (LAG) feature, developed based on the human eye visual system to avoid downsampling the image resolution to fit the network. In addition, the dual transfer learning (DTL) strategy was implemented to train the AI model, with connections found between WLI and LCI. The network achieved an accuracy of 99.18% and 97.12% in recognizing GIM and CAG, respectively, which exhibits state-of-the-art performances. Moreover, the network was compared against four endoscopists with experience whose performance was inferior to the suggested model [16].

Endoscopy images of the stomach are quite alike in shape and color, which could cause the main DL models to underperform. Chong et al. created the Multi-scale with Attention-net (MWA-net) network that could improve the accuracy of diagnosing CAG. Using 5159 endoscopic images of the gastric antrum, MWA-net was compared with well-known CNNs such as ResNet, DenseNet, and Inception v3. The MWA-net attained the best performance in sensitivity, specificity, and accuracy. Moreover, the network was compared with a beginner and an expert group of endoscopists. The expert group showed a better performance in contrast with the beginner group, while the network outperformed the expert group in terms of sensitivity (90.19% vs. 86.00%; *p* < 0.05), specificity (94.51% vs. 88.26%; *p* < 0.05), and accuracy (92.13% vs. 87.01%; *p* < 0.05). Furthermore, the heat map generated by the attention module of this DL network could offer an outline for gastroscopists to discover the atrophied area effortlessly. Implementing this model could reduce the workload of clinicians by decreasing the time spent on analyzing images from 26.5 min in the beginner group and 25.7 min in the expert group to 0.71 min with MWA-net [17].

Luo et al. conducted a two-center study testing two DL models to correctly identify gastric antrum atrophy and its severity and CAG. ResNet-50 was chosen after testing and comparing multiple architectures by exhibiting the best performances. This study was based on the usual WLI broadly used in public hospitals. CAG arises in all stomach parts; thus, the images were obtained from the fundus, body, angle, and antrum. The study revealed that model 1 achieved a high accuracy in recognizing gastric antrum atrophy in both internal test sets—90.2%—and external test sets—89%. However, the accuracy of identifying the severity of gastric antrum atrophy was lower in the internal test set—77.3%—and considerably lessened in the external test set—59%. The group discovered that when using three test sets, the second model efficiently diagnosed CAG in both hospitals. The study’s final experiment evaluated model 1 and its ability to detect gastric antrum atrophy against three skilled endoscopists, with similar results [18].

Most studies surrounding the idea of AI-based diagnosis of CAG have a retrospective design, with endoscopic images sorted in advance. Zhao et al. designed a prospective cohort study that analyzed the performance of a DL model of diagnosing CAG using real-time video monitoring. The U-NET model was trained and tested using 5290 endoscopic images acquired utilizing the narrow-band imaging technique (NBI). Testing the network showed an accuracy of 92.63% in diagnosing CAG. Then, they compared the AI network with a group of gastroscopists, considering the severity and the number of atrophy sites, along with the degree of intestinal metaplasia, inflammatory activity, and HP infection of the atrophy sites. The study cohort comprised 268 patients, and by using a paired design, the patients in the DL group and the endoscopist group were identical. The results indicated that the AI model ameliorated the diagnosis rate of endoscopic CAG compared with that of endoscopists. Additionally, the DL model identified more cases of moderate and severe CAG than the gastroscopists’ diagnoses. Moreover, the U-NET model discovered more atrophy areas, and consequentially, the number of biopsies was reduced. This research suggests that computer-aided diagnosis (CAD) could aid clinicians in detecting and diagnosing precancerous lesions swiftly, and in reducing the number of biopsies needed to diagnose [19].

Zhao et al. conducted a prospective case-control study to evaluate the DL real-time video assessment model further. Thus, more patients were added to the previous cohort, and propensity score matching (PSM) was used to identify patients with similar characteristics. Taking the histopathological diagnosis as the gold standard, the study evaluated the AI model’s consistency with the pathological diagnosis in the two groups—CAG and chronic non-atrophic gastritis (CNAG)—with the results indicating that the DL network had better accuracy compared with the human experts. Moreover, a subgroup analysis was conducted to assess the diagnosis performances of the DL model of mild, moderate, and severe CAG. The outcomes were consistent with the previous study, exhibiting superior performances with AI [20].

Lin et al. investigated whether CNNs could concomitantly identify CAG and GIM on WLI. Thus, TResNet, a DL model, was developed, trained, validated, and tested using 7037 endoscopic images, with 2899 labeled AG and GIM. The outcomes indicated that the AUCs for correctly identifying AG and GIM were 98.3% and 99%, respectively. Additionally, the DL model was tested against a group of endoscopists, who analyzed the images and provided a diagnosis of AG or GIM. Results showed that compared with the CNN, the endoscopists presented a relatively low sensitivity, varying from 35.2 to 51.7% for recognizing AG and 28.2–47.3% for GIM. This signifies that the DL network could better exclude the presence of AG and/or GIM than the endoscopists in clinical practice. Because of this, the number of biopsies for screening gastric cancer could be reduced drastically. Furthermore, the AI model could aid clinicians by generating class activation maps outlining abnormal areas [21].

Zhang et al. investigated whether DL algorithms could improve the recognition and diagnosis of CAG and its severity. Because atrophic gastritis mainly affects the antrum, 5470 endoscopic images of the entire gastric antrum were used in this study. DenseNet was the CNN selected to execute this research due to the high potential of identifying CAG. It was trained by utilizing five-cross validation to ensure the model was reliable, and then it was tested to assess its performance. The AI model’s sensitivity, specificity, and accuracy were 94.58%, 94.01%, and 94.24%, respectively, better than those of expert endoscopists. The accuracy of diagnosing mild, moderate, and severe gastritis was 93%, 95%, and 99%, respectively. Class activation mapping (CAM) was used to generate heatmaps for each endoscopic image, which helped highlight the areas of gastric atrophy [22].

Horiuchi et al. assessed if a CNN could improve distinguishing between early gastric cancer (EGC) and CAG using magnifying endoscopy with narrow-band imaging (ME-NBI). The group trained the DL model GoogLeNet with 1492 EGC and 1078 gastritis images and then tested it with an external data set that included 151 EGC and 107 gastritis images. The overall accuracy of this AI model for correctly identifying the two stomach diseases was 85.3%. In addition, the sensitivity and negative predictive value were 95.4% and 91.7%, respectively. The specificity for this model was 71%, and that was due to localized atrophy and the presence of intestinal metaplasia on the endoscopic images. In clinical practice, this CNN could aid endoscopists in differentiating EGC and gastritis using ME-NBI by delivering a response swiftly with good accuracy [23].

### 3.2. Artificial Intelligence Based on Pathology Slides

We found four studies evaluating AI capability in identifying gastritis on pathology slides, as illustrated in Table 2.

Franklin et al. studied whether a DL model could diagnose and distinguish between two inflammatory gastric diseases: *Helicobacter pylori* gastritis (HPG) and Autoimmune Gastritis (AuG). The group trained and tested HALO-AI with 187 images of gastric tissue fragments of both HPG and AuG. The outcomes of the HALO-AI were equal to those of two expert gastrointestinal (GI) pathologists, which showed 100% concordance with the gold standard diagnosis. Two non-GI trained surgical pathologists and two pathology residents competed against the DL model, but their results were inferior, with 86% and 57% concordance, respectively. Although HPG mainly affects the antrum while the body and the fundus are less altered, HALO-AI could assign the correct diagnosis even if all the tissue fragments were in the image. This is, for the most part, possible because the DL integrated a spectrum of inflammation into its learning [24].

Lin et al. developed a two-tier, deep learning-based model for diagnosing HP gastritis using 885 WSIs only labeled as positive or negative. The model had an AUC of 0.974 (0.955–0.993) with a sensitivity of 0.933 and a specificity of 0.901, compared to pathologists with a sensitivity of 0.933 and a specificity of 0.842. An auxiliary model was also developed using 824 areas with HP in 9 positive WSIs and 446 negative WSIs that could localize HP with a precision of 0.58 [25].

A study using 2725 WSIs from 545 patients (436 patients with 2180 WSIs for discovery and 109 patients with 545 WSIs for validation) developed a model (GasMIL) to diagnose and grade GA and IM. GasMIL had a high performance in all data sets, in the training set obtaining AUCs of 0.97, 0.981, 0.884, 0.877, 0.79, and 0.79 for inflammation, activity, IM, atrophy, OLGIM, and OLGA. GasMIL was also compared with 10 pathologists, obtaining better results than most of them. When GasMIL assisted pathologists, there was a statistically significant difference in IM AUC, sensitivity and weighted kappa, and atrophy specificity, but no improvement in IM specificity, atrophy AUC, sensitivity and weighted kappa, OLGIM, and OLGA [26].

Ma et al. developed an attention-based multi-instance multilabel learning network (AMMNet) to diagnose gastritis indicators and provide interpretable labels using data from 1096 patients. AMMNet performed well, with 0.93 AUC for evaluating activity, 0.97 AUC for GA, and 0.93 AUC for IM. Junior pathologists had lower false-negative rates when assisted by AMMNet. The model also reduced the time spent per slide from 5.46 min to 2.85 min [27].

Ba et al. tested AI’s efficiency in diagnosing different types of chronic gastritis from a histological point of view using whole-slide images (WSIs). A team of two senior pathologists independently analyzed 1250 slides to give the final diagnosis as the reference gold standard. A total of 1128 WSIs with the three kinds of gastritis—chronic superficial gastritis (CSuG), chronic active gastritis (CAcG), and CAG—and 122 with normal gastric mucosa were used to train, validate, and test the Deep-Lab v3 network. The results showed that the AI model obtained AUCs of 88.2%, 90.5%, and 91% for CSuG, CAcG, and CAG, respectively. The specificity and sensitivity of this model for diagnosing CSuG were 100% and 79%, for CAcG were 82.9% and 98.5%, and for CAG were 99.2% and 95.2%. Altogether, the accuracy of the DL network for the three distinct types of chronic gastritis was 86.7%. By emphasizing the regions of interest on a biopsy slide, this AI tool could aid pathologists in making a quicker diagnosis [28].

Ma et al. elaborated a study analyzing AI’s potential of recognizing the three main states of the stomach which could generate the main path of gastric carcinogenesis: normal mucosa, chronic gastritis, and intestinal-type gastric cancer. A total of 763 WSIs were separated into image patches and then formatted through stain normalization to standardize the colors in the histopathological images and data augmentation to create multiple samples. Inception v3 was chosen as the suitable AI model to execute the experiment. The best accuracy for classifying the three classes was 94.5%. A cancer-likelihood map was generated for each WSI by combining the heat maps from each image patch. After that, the DL network processed WSI characteristics, and 44 features were extracted from the maps. By employing gradient-weighted class activation mapping (Grad-CAM) and saliency mapping, the main elements for each gastric lesion were emphasized. The group also conducted a survival analysis to demonstrate DL’s utility in predicting outcomes. By collecting follow-up data and training the AI model with the clinicopathological features and the 44 features extracted from the heatmaps, the DL model had a prediction accuracy of 97.4% [29].

Steinbuss et al. examined the effectiveness of CNNs to identify different subtypes of gastritis using pathology slides. Taking etiology as the main criteria, gastritis can be classified as autoimmune (type A), bacterial (type B), and chemical (type C). Pathologists marked images with gastritis as low inflammation (LI) or severe inflammation (SI). They were divided into two groups depending on the region where they were taken from: the antrum or the corpus of the stomach. The Xception model was selected as the CNN to carry out this hypothesis, and two classifiers were trained, validated, and tested using randomly assigned image patches from the histopathological slides. The results revealed an accuracy of 85% for the antrum classifier and 56% for the corpus one. The main downside of this study was the small dataset, with only 135 pathology images of all three types of gastritis [30].

### 3.3. Artificial Intelligence Based on Double-Contrast UGI Barium X-rays

The main studies investigating the accuracy of AI in diagnosing gastritis using double-contrast UGI barium X-rays are exhibited in Table 3.

A study performed by Kanai et al. researched if AI could detect CAG on contrast gastric X-ray images (GXIs). Inception v3 was utilized as the CNN to run this experiment. The group used 815 GXIs to train, fine-tune, and examine the network. To be more efficient and avoid manually annotating the stomach region on each X-ray, they formed two training groups: the manual annotating group (MAG), with a small number of GXIs, and the automatic annotating group (AAG). The X-rays were divided into positive patches (P) with CAG, negative patches (N) with CNAG, and unrelated patches (U) from outside the stomach. Firstly, they tested the model’s ability to automatically annotate the stomach regions, which was estimated to be highly accurate. Secondly, the model was assessed for CAG recognition. The outcomes revealed that the detection performance is higher for MAG and AAG together than for MAG alone, which shows the efficiency of automatically annotating the stomach regions. The experiment was conducted only with the AAG to verify further the skill to reduce the workload by automatization, and the harmonic mean was 96.5% [31].

Li et al. conducted a study to test their semi-supervised learning method for recognizing chronic gastritis using GXIs. This study is based on tri-training, which implies training three models by augmenting each model’s training set with the other two models. A total of 815 GXIs were cropped into patches and divided into three groups: gastritis, non-gastritis, and irrelevant. Additionally, for data augmentation, this study uses Between-Class (BC) learning, which generated a better performance and gave the models a better generalization capability. The three CNNs used were ResNet, DenseNet, and the simplest network. The outcomes of this analysis showed high precision for diagnosing chronic gastritis with a restricted number of annotated images. The best results were achieved using 100 annotated images with a sensitivity of 92.2%, a specificity of 90.7%, and a harmonic mean of 91.4% [32].

Togo et al. developed a deep CNN to detect gastritis using double-contrast upper gastrointestinal barium X-rays (UGI-XRs). This method was compared with the ABC(D) stratification, which utilizes serum levels of pepsinogen (PG) and H. pylori IgG antibodies to assess gastric cancer risk. A total of 815 patients were included in the study, and UGI-XRs were taken for each one and obtained from eight different positions, resulting in 6520 images. The group created a deep CNN model based on image patches, which was then programmed to identify gastritis regions on all patches. The final, refined model was able to differentiate the areas of interest from the non-gastritis ones. Results indicated that the team’s method had high sensitivity, specificity, and harmonic mean levels of 96.2%, 98.3%, and 97.2%, respectively. Moreover, the outcomes were similar to those of ABC(D) stratification. The combined use of this AI model and the serology-based test could give more precise information about the risk of gastric cancer [33].

Li et al. developed a model that used 815 gastric X-rays (240 positive and 575 negative; 100 positive and 100 negative images were used for the training set and the rest for the test set) to execute explicit self-supervised learning and learn discriminative representations. The model had a better performance than five self-supervised learning methods (SimSiam, BYOL, PIRL-jigsaw, PIRL-rotation, and SimCLR) and three previous models, obtaining a harmonic mean score of sensitivity and specificity of 0.875, 0.911, 0.915, 0.931, and 0.954 when using the data from 10, 20, 30, 40, and 200 patients [34].

### 3.4. Artificial Intelligence Based on Clinical and Serological Findings

Lahner et al. evaluated if advanced statistical methods, such as artificial neural networks (ANNs) and linear discriminant analysis (LDA), could help diagnose atrophic body gastritis (ABG) while employing only clinical and biochemical data. They included 350 patients, 263 with ABG and 87 without ABG, and for each patient, a questionnaire with 22 items was completed, resulting in 37 variables in the data set. Among the anamnestic and clinical variables, biochemical ones like fasting gastrin levels, pepsinogen I levels, and antibodies against anti-parietal cells were evaluated. They conducted five experiments. The first one was tested with all the variables from the data set, and the second used the training and testing algorithm alongside the input selection system, which reduced the number of variables to the most informative ones. The third one was performed using the eight items having the most predictive power for recognizing ABG. In the fourth experiment, the serological variables were excluded from the ones utilized in the previous experiment, and in the fifth test, only the serological variables were used. Results illustrated the highest performances during the second experiment, with an overall accuracy of 98.8% for the ANN model and 96.75% for the LDA one [35].

## 4. Discussion

AI applications have been gradually tested and implemented in various fields during the digital era to simplify the working methodology. Physicians could benefit from digitalization and automatization in the medical domain by reducing their workload and providing superior healthcare services. Most studies analyzing AI functions to detect gastritis showed a high level of accuracy similar to or greater than that of human experts. Taking the histopathological result as the gold standard, machine learning models have been trained, validated, and tested to recognize gastric inflammation with various approaches: endoscopic images, pathology slides, gastric X-rays, and clinical–serological parameters.

The DL networks employed on endoscopic images were represented entirely by CNNs, one of the most popular networks for image classifications, handling large amounts of data and generating highly accurate outcomes. In most studies, gastroscopy images were acquired with the WLI mode, thereby simulating the environment in public hospitals. This could ensure an easier replication of the DL model across all healthcare facilities. Another strength identified among these studies was the networks’ heat maps, highlighting the gastric mucosa’s suspicious areas, guiding the endoscopists, and shortening the procedure and diagnosis duration. However, a few limitations were found. Firstly, only three studies were prospective, assessing in real-time and automatically during gastroscopy, with the rest being retrospective. Secondly, nearly all investigations, except two, limited their database to only a center, which could be a bias-generating factor [12,13,14,15,16,17,18,19,20,21,22,23].

Digital pathology slides must be separated into image patches as CNNs can scan only small sizes. Research on AI applications based on histopathological findings has demonstrated that CNNs could be used as a screening method to automatically prefilter and emphasize the relevant areas before the pathologist’s review. As in the case of endoscopic images, heatmaps can be generated for guidance, plus cancer likelihood maps, which can stratify the risk of developing cancer. The limitations previously presented were also found in studies concerning pathology slides. The small database inconvenience is mostly solved through data augmentation [24,25,26,27,28,29,30].

AI applications based on double-contrast UGI barium X-rays have depicted promising results for recognizing chronic gastritis. This could reform the screening process in countries with a high prevalence of gastric cancer by being the first step for classifying patients depending on the risk for neoplasia [31,32,33,34]. As ANNs and LDAs rely on clinical and serological variables, these AI models can support the diagnosis decision and select the patients who need to undergo more extensive and invasive procedures, saving time and medical resources [35].

In the same context, a systematic review and meta-analysis conducted by Shi et al. investigated eight studies surrounding AI applications based on endoscopy for diagnosing chronic atrophic gastritis. The sensitivity of AI for recognizing CAG was 94% (95% CI: 0.88–0.97), while the specificity was 96% (95% CI: 0.88–0.98) and the AUC was 98% (95% CI: 0.96–0.99). The research showed that the accuracy of AI in identifying CAG was greater than that of endoscopists [36]. In addition, Luo et al. screened the literature for relevant research on AI systems recognizing EGC, CAG, and HP infection. The review depicted the effectiveness of machine learning in supporting the endoscopic detection of gastric neoplasia, predicting the extent of invasion, providing precise outlining of the tumor margins, and recognizing CAG and HP infection [37].

The systematic review we carried out has numerous strengths. First, we examined all the accessible techniques for an automatic diagnosis of gastritis by utilizing AI-based methods involving gastric endoscopy, pathology studies, UGI X-rays, and biological parameters. Second, the subject of this systematic review is extremely important since it may help clinicians diagnose gastritis more accurately. Despite being one of the most common gastrointestinal disorders, many cases of gastritis are either incorrectly or never identified because of a variety of circumstances, including vague symptoms, late presentation to the healthcare professional, inaccurate diagnosis, and rushed gastroscopies. Therefore, implementing AI might lighten the strain on endoscopists and pathologists, enhance the skills of new residents, speed up diagnosis, and minimize human mistakes.

Our systematic review has several limitations, which should be mentioned. The first one is that most studies come from a single medical facility. Multi-center studies are recommended to address this drawback, as they will increase the accuracy of diagnosing gastritis and the capacity to generalize the results more easily. Secondly, most studies are retrospective, and thirdly, they vary concerning the methods used, design, evaluation, and results, and this inconsistency complicates comparing the outcomes.

Future research should concentrate on a few crucial areas to improve AI for diagnosing gastritis. First, the comprehensiveness of diagnostic tools may be enhanced by integrating multimodal data, which combines endoscopic pictures, clinical records, and patient demographics. Assessing AI systems’ long-term efficacy and dependability in practical environments requires longitudinal research. Understanding the benefits and applicability of current models will be possible through cross-validation and benchmarking versus conventional techniques.

Substantial challenges, such as ethical issues, regulation, governance, security, practicability, and general approval, need to be overcome before implementing AI in healthcare. However, cooperation between physicians and AI technologies is necessary to achieve excellent medical results because AI cannot operate independently or substitute human intelligence.

Lastly, considering the future problems addressed, reviewing and comparing different AI-based methods for the automatic diagnosis of gastritis requires additional assessment. Establishing an elaborate but definite list of criteria for future analyses portrays a practical perspective to settle this intricate matter.

## 5. Conclusions

AI provides a compelling trajectory toward further research involving gastrointestinal diseases. Mainly, feasible uses consist of prompt and automated pathology identification, personalized risk assessment, treatment optimization, and improved prognostic outcome. While acknowledging the various difficulties linked with implementation, the likelihood of incorporating AI seamlessly into the healthcare system remains encouraging. This systematic review offers an extensive investigation of the efficacy of AI applications for diagnosing gastritis. AI-based technologies can now automatically diagnose using images provided by UGI gastroscopy and digital pathology. Nevertheless, more extensive, multicentric studies are needed to validate and support the current findings. DL models exhibited high levels of accuracy, sensitivity, and specificity while assessing the diagnosis, staging, and risk of neoplasia for different types of gastritis, results that are comparable to those of human experts.

## Figures and Tables

**Table 1:** Studies evaluating AI’s ability to diagnose gastritis using gastroscopy images and/or videos.

Study	Year of Publication	Country	Study Design	Validation Method	Number of Images/ Videos	Diagnosis	Main Findings
Shi et al. [12]	2023	China	Retrospective	Internal and External	10,961 images and 118 videos	CAG	The GAM-Efficient net showed an accuracy of 93.5% in diagnosing CAG in images and 92.37% in videos.
Jhang et al. [13]	2023	Taiwan	Retrospective	Internal	912 images	CGI	The model performed better than the five models it was compared to, with an AUC of 0.984, accuracy of 0.957, sensitivity of 0.938, and specificity of 0.962.
Tao et al. [14]	2024	China	Retrospective	Internal and External	869 images and 119 videos	CAG	The model performed better in the image competition except compared to the expert endoscopists. The model performed better than all groups of endoscopists in the video competition and CAG stratification.
Zhao et al. [15]	2023	China	Prospective	Internal	5290 images	CAG, OLGA	The model performed better than endoscopists for all parameters (sensitivity, specificity, PPV, NPV, accuracy rate, Youden index, odd product, LR+, LR−, AUC, and kappa) and for diagnosing OLGA.
Yang et al. [16]	2023	China	Retrospective	Internal	21,240 images	CAG and CIM	The AI model attained state-of-the-art performances with an accuracy of 99.18% and 97.12% in identifying GIM and CAG, respectively.
Chong et al. [17]	2022	China	Retrospective	Internal	5159 images	CAG	The sensitivity and specificity achieved by MWA-net were 90.19% and 94.15%, which were greater than those of an expert group of endoscopists.
Luo et al. [18]	2022	China	Retrospective	Internal and External	10,593 images	CAG	The deep learning model’s performance to recognize CAG and its severity was similar to that of a group of gastroenterologists.
Zhao et al. [19]	2022	China	Prospective	Internal	5290 images	CAG	The U-NET model identified more cases with moderate and severe CAG in contrast with a team of gastroscopists.
Zhao et al. [20]	2022	China	Prospective	Internal	5290 images	CAG	By taking the histopathological diagnosis as the gold standard, the real-time video assessment model showed superior results compared to human experts.
Lin et al. [21]	2021	China	Retrospective	Internal	7037 images	CAG and GIM	The CNN network, TResNET, attained high performances in recognizing AG and GIM.
Zhang et al. [22]	2020	China	Retrospective	Internal	5470 images	CAG	DenseNet exhibited high levels of accuracy in diagnosing mild, moderate, and severe gastritis: 93%, 95%, and 99%, respectively.
Horiuchi et al. [23]	2019	Japan	Retrospective	Internal	2828 images	EGC and CAG	The overall accuracy of this CNN model of recognizing the two stomach pathologies was 85.3%.

CAG: chronic atrophic gastritis; CGI: corpus-predominant gastritis index; OLGA: operative link on gastritis assessment; GIM: gastric intestinal metaplasia; EGC: early gastric cancer; CNN: convolutional neural networks; AG: atrophic gastritis.

**Table 2:** Studies analyzing AI’s effectiveness in diagnosing gastritis using pathology slides.

Study	Year of Publication	Country	Study Design	Validation Method	Number of Pathology Slides	Diagnosis	Main Findings
Franklin et al. [24]	2022	USA	Retrospective	Internal	187	HPG and CAG	The results of the AI model were equal to those of two expert pathologists, which illustrated 100% concordance with the gold standard diagnosis.
Lin et al. [25]	2023	China	Retrospective	Internal	885	HPG	The model had 0.974 AUC, 0.933 sensitivity, and 0.901 specificity, compared to pathologists with 0.933 sensitivity and 0.842 specificity.
Fang et al. [26]	2023	China	Retrospective	Internal and External	2725	GA, IM	The model had a high performance, obtaining better results than most pathologists, but when assisting them only improved a few parameters (IM AUC, sensitivity and weighted kappa, and atrophy specificity).
Ma et al. [27]	2024	China	Retrospective	Internal	1096 patients	GA, IM	The model had a high performance, with 0.93 AUC for evaluating activity, 0.97 AUC for GA, and 0.93 AUC for IM. It also reduced the time spent on each slide from 5.46 min to 2.85 min.
Ba et al. [28]	2021	China	Retrospective	Internal	1250	CSuG, CAcG, and CAG	For the three different types of chronic gastritis, the deep learning network showed an accuracy of 86.7%.
Ma et al. [29]	2020	China	Retrospective	Internal	763	Normal mucosa, CAG, GC	The highest accuracy for recognizing the three states of the gastric mucosa was 94.5%. After collecting follow-up data and fine-tuning the AI model with more features, the DL model had a survival prediction accuracy of 97.4%.
Steinbuss et al. [30]	2020	Germany	Retrospective	Internal	135	Autoimmune, bacterial, and chemical gastritis	Diagnosis of bacterial gastritis using a deep learning model was performed with a sensitivity of 100% and a specificity of 93%.

HPG: *Helicobacter pylori* gastritis; CAG: chronic atrophic gastritis; GA: gastric atrophy; IM: intestinal metaplasia; CSuG: chronic superficial gastritis; CAcG: chronic active gastritis; GC: gastric cancer.

**Table 3:** Studies assessing AI’s potential for diagnosing gastritis using double-contrast upper gastrointestinal barium X-rays.

Study	Year of Publication	Country	Study Design	Validation Method	Number of X-rays	Diagnosis	Main Findings
Kanai et al. [31]	2020	Japan	Retrospective	Not specified	815	CAG	The AI model showed high performance in recognizing CAG, even when the number of manually annotated X-rays was limited.
Li et al. [32]	2020	Japan	Retrospective	Internal	815	CAG	High precision in diagnosing chronic gastritis was attained with a restricted number of annotated images. Using 100 annotated images, the best results were achieved with a sensitivity of 92.2% and a specificity of 90.7%.
Togo et al. [33]	2018	Japan	Retrospective	Internal	6520	CAG	The sensitivity, specificity, and harmonic mean were 96.2%, 98.3%, and 97.2%, respectively, which were similar to those obtained with the ABC (D) stratification.
Li et al. [34]	2023	Japan	Retrospective	Internal	815	Gastritis	The model had a harmonic mean score of sensitivity and specificity of 0.875, 0.911, 0.915, 0.931, and 0.954 when using data from 10, 20, 30, 40, and 200 patients.

CAG: chronic atrophic gastritis.

## Abbreviations

**Abbreviation**	**Definition**
AAG	Automatic Annotating Group
ABG	Atrophic Body Gastritis
AG	Atrophic Gastritis
AI	Artificial Intelligence
AuG	Autoimmune Gastritis
CAD	Computer-Aided Diagnosis
CAG	Chronic Atrophic Gastritis
CAcG	Chronic Active Gastritis
CAM	Class Activation Mapping
CGI	Corpus-Predominant Gastritis Index
CNAG	Chronic Non-Atrophic Gastritis
CNN	Convolutional Neural Networks
CSuG	Chronic Superficial Gastritis
DL	Deep Learning
DTL	Dual Transfer Learning
EGC	Early Gastric Cancer
GAM	Global Attention Mechanism
GC	Gastric Cancer
GIM	Gastric Intestinal Metaplasia
HP	Helicobacter Pylori
HPG	Helicobacter Pylori Gastritis
LAG	Local Attention Grouping
LCI	Linked-Color Imaging
LI	Low Inflammation
MAG	Manual Annotating Group
ME-NBI	Magnifying Endoscopy with Narrow Band Imaging
NBI	Narrow-Band Imaging
OLGA	Operative Link on Gastritis Assessment
PG	Pepsinogens
PSM	Propensity Score Matching
SI	Severe Inflammation
UGI	Upper Gastrointestinal
WLI	White Light Imaging
WSIs	Whole-Slide Images
